# A Correlation Study between Two Adjacent Same-Meridian Acupoints after Laser-Needle Acupuncture with Optical Coherence Tomography and Diffuse Reflectance Spectra

**DOI:** 10.1155/2016/7482587

**Published:** 2016-11-20

**Authors:** Xiuli Wu, Huajiang Wei, Zhouyi Guo, Yirong Ni, Zhiming Liu, Hongqin Yang, Shusen Xie, Lianpeng Zhou

**Affiliations:** ^1^MOE Key Laboratory of Laser Life Science & SATCM Third Grade Laboratory of Chinese Medicine and Photonics Technology, College of Biophotonics, South China Normal University, Guangzhou 510631, China; ^2^Key Laboratory of Optoelectronic Science and Technology for Medicine of Ministry of Education of China, Fujian Normal University, Fuzhou 350007, Fujian, China

## Abstract

This study is to investigate the correlations among Sanjian (LI3), Hegu (LI4), and Yangxi (LI5) acupoints and their corresponding nonacupoints on the Yangming Large Intestine Meridian of Hand before and after laser irradiation using optical coherence tomography (OCT) and diffuse reflectance spectra. The experiment was conducted on 10 healthy people. A 658 nm laser with 50 mW output power was used for irradiating LI4, LI5 acupoints and their corresponding nonacupoints. As to LI4 acupoint with laser irradiation for duration of 15 or 45 minutes, the OCT backscattered light intensities of LI4 and LI5 acupoints increased significantly, and the reflectance intensities (RIs) of the LI3, LI4, and LI5 acupoints decreased significantly. As to LI5 acupoint with laser irradiation for duration of 15 or 45 minutes, the changes of OCT backscattered light intensities of the corresponding irradiated acupoint and LI4 acupoint increased significantly, and the RIs decreased significantly. However, the OCT backscattered light intensities and RIs for their nonacupoints were almost not changed. The results show that an association exists between two adjacent same-meridian acupoints on the same meridian after laser-needle acupuncture to some extent.

## 1. Introduction

Meridians play a very important role in the traditional Chinese medicine. They are supposed to channel some kind of substance, energy, or information that is called “qi” in the traditional Chinese medical literature. Experts and scholars from China and abroad did a lot of researches on meridians for recent decades and proposed many assumptions about the meridian theory. Acupuncture hypothesis in the traditional Chinese medicine has achieved a great success in clinical applications and has also begun to gain acceptance in the West [1–3]. In recent years, laser acupuncture with characteristics such as painless, noninvasive, and low risk of infection compared with the traditional acupuncture have appeared and taken into clinical applications where a laser beam is used to irradiate acupoints [4]. Although the clinical effectiveness of laser acupuncture has been confirmed, its mechanism remains unclear. Many studies have reported that the therapy effects of laser acupuncture are very similar to the traditional acupuncture for a lot of diseases and better for some diseases [5–8]. In particular, the risk of contracting AIDS or other infectious diseases is absent. Moreover, the technique does not need sophisticated instruments, which makes it more widely used in developing countries [9–11]. The mechanism of laser acupuncture was researched by all kinds of modern techniques in recent years, such as the electricity, ultrasound, and magnetic resonance imaging (MRI) [11–15]. Among them, the electricity technique was the most widely used technique [16–19]. These methods can reveal the existence of meridian and its properties to certain extent. However, these techniques have their own specific shortcomings. For example, the resolutions of the ultrasound and MRI are not high, which limits their applications in laser acupuncture mechanism exploration, although they are noninvasive [4]. To the best of our knowledge, optical techniques can obtain high-resolution physical structures of tissues and their functional information noninvasively. Some studies about the optical techniques were reported in recent years. For example, Huang et al. [4] had studied changes of optical attenuation coefficients of acupoints during laser acupuncture by optical coherence tomography (OCT). Yang et al. [20] had studied differences in optical transport properties between human meridian and nonmeridian. Chen et al. [21] have studied the light wave transport characteristics variation along the pericardium meridian under different pressures. The optical signal on the meridian is stronger than the nonmeridian. The optical signal attenuates slower when it transported along meridians. The human meridians may be the good pathway for light waves with certain wavelengths. That is to say light waves with certain wavelengths can transport and be blocked along meridian direction, when they irritate the meridians, or acupoints after excluding the influence of human anatomy structure, which verified again the objective existence of meridians transporting and the possibility of blocking from the optical view. In order to discuss specificity of acupuncture on the response of qi and blood in human body, Jiang et al. [22] compared the diffuse reflectance of Taichong (LV3), Taibai (SP3), and Chongyang (ST42) before, during, and after menstruation. The experimental data suggested that light in a certain wavelength may propagate along the pericardium meridian compared to the nonmeridian direction. Zhong et al. [23] used OCT images to distinguish human acupoint from nonacupoint after irradiation with laser* in vivo*. The results of the study showed significant differences between Laogong acupoint and nonacupoint tissues in the OCT signal intensity. These studies discovered the properties of acupoint and meridian by laser irradiation. However, they did not research the characteristics of optical spectrum between adjacent acupoints on the same meridian by laser irradiations.

**Figure 1:**
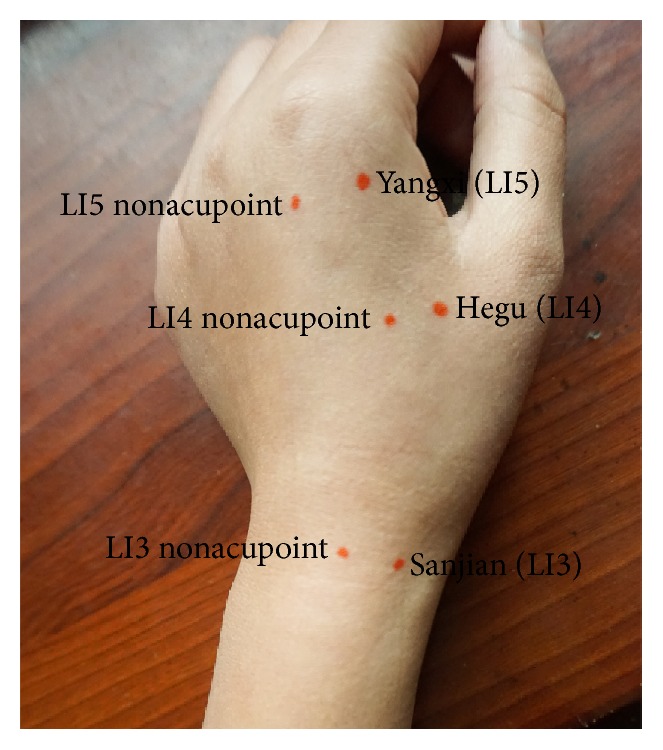
The locations of acupoint and nonacupoint. “Red dot” shows the location of nonacupoint of the corresponding acupoint.

LI3, LI4, and LI5 acupoints are located on the Yangming Large Intestine Meridian of Hand (Figure 1). The distance between acupoint and its corresponding nonacupoint was 10 mm on the ulnar side of corresponding acupoint. LI4 is located on the highest spot of the muscle when the thumb and index fingers are brought close together. LI4 is clinically used for stress, facial pain, headaches, toothaches, and neck pain. The ancient traditional Chinese medicine text includes a wide range of indications for LI4 from headaches and constipation to general pain and delayed labor. LI4 is a point that has been extensively studied through randomized controlled trials and clinical research. Recent studies from the Journal of Orofacial Pain showed that the stimulation of LI4 significantly reduced myofascial pain of the jaw muscles [24]. A recent Cochrane systematic review on acupuncture in migraine and tension-type headaches suggests stimulation of LI4 acupoint as an effective and valuable option for alleviating migraines and tension-type headaches [25]. With a loose fist, LI3 is located on the radial side of the index finger, in the depression proximal to the second metacarpophalangeal joint. LI3 is clinically used for swollen, sore throat, fever, and toothaches. LI5 is situated in the transverse crease of the radial side of the wrist, between the hollow of the tendon of the extensor pollicis longus and brevis. LI5 is used to treat pain in the wrist, headache, toothache, and swollen and sore throat.

Zhou et al. [26] studied the effects of titanium dioxide nanoparticles coupled with diode laser on optical properties of* in vitro* normal and cancerous human lung tissues studied with OCT and diffuse reflectance spectra. In this paper, a scheme was designed to explore the differences of optical spectra and OCT backscattered light intensities between the LI4 acupoint and its adjacent acupoints after a 658 nm laser irradiation* in vivo*. The experimental results showed that an association exists between adjacent acupoints on the same meridian to some extent. This method would be very helpful in further research of specificity of acupoint and clinical applications for laser-needle acupuncture.

## 2. Materials and Methods

### 2.1. Participants

Ten healthy participants (5 males, 5 females) were recruited from South China Normal University [mean age ± standard deviation (SD), (22.0 ± 2.0) years]. The subjects had no history of chronic disease and were healthy at the time of enrollment. The positions of acupoints were determined according to the National Standard of China “Location of Acupoints” (GB 12346-90) [27]. The distance between acupoint and its corresponding nonacupoint was 10 mm on the ulnar side of corresponding acupoint. The experimental protocol was approved by the Laboratory of Photonic Chinese Medicine, Ministry of Education Key Laboratory of Laser Life Science and Institute of Laser Life Science, South China Normal University. All subjects agreed to participate in the study and provided written informed consent.

### 2.2. Experimental Setup

**Figure 2:**
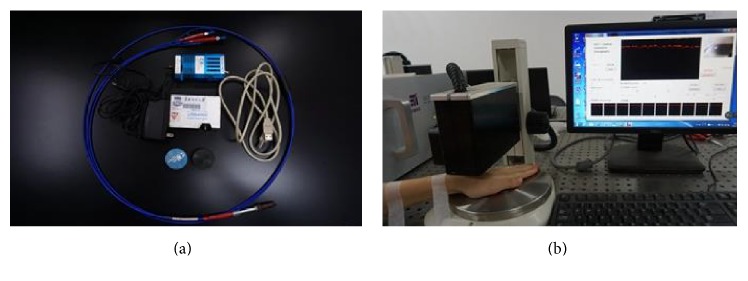
Experimental setups: (a) optical fiber spectrometer; (b) commercial spectral domain OCT (SD-OCT) system.

The reflectance spectra of acupoints were measured using a commercial optical fiber spectrometer (Figure 2(a)) (Ocean Optics, USA, model: USB 4000) in the spectral range 200 to 1100 nm. The tungsten halogen light source (Ocean Optics, USA, model: LS-450) served as a source of light. The fiber-optic probe (Ocean Optics, USA, model: USB 4000) consisting of seven fibers with the internal diameter 400 *μ*m and the numerical aperture 0.2 was used in the measurements. The central fiber was served for collecting the diffuse reflected radiation, while the surrounding six fibers were used for the illumination of the sample. The probe was placed at the distance of 2 mm from the sample surface and registered the signal, averaged over the area of the radiation collection. The spectra were calibrated against a DR standard of BaSO_4_ with a smooth surface. All spectra measurements were recorded with the integration time set to 100 ms. The measurements were recorded and stored into a computer for the further postprocessing [26].

The experiments were implemented using a commercial spectral domain OCT (SD-OCT) system (Figure 2(b)) (Shenzhen MOPTIM Imaging Technique Co., Ltd., China) working at the central wavelength 830 ± 40 nm with an optical power of 5 mW, a maximum image depth of 1.6 mm, a signal-to-noise ratio of 120 dB, and a length of scanned area of 3 mm. The SD-OCT system, determined by the focal spot size of the probe beam, provides an axial resolution of 12 *μ*m and a transverse resolution of 15 *μ*m in free space. Two-dimensional (2D) images are obtained by scanning the incident beam over the sample surface in the lateral direction and in-depth (A-scan) scanning by the interferometer. OCT images were acquired over a period of 240 min in 1-minute intervals to record transient changes of optical tissue properties.

### 2.3. Experimental Procedures

The experimental measurements were performed in a dark room, and the room temperature was kept at (25.0 ± 5.0)°C and the relative humidity at 65%  ±  15% during the measurements. The laser irradiation points were cleaned and labeled before the experiment with the help of an acupuncturist. To reduce the measurement error induced by the movement of the subject arm, each subject was made to sit on a chair with arms fixed in a comfortable position using the cellophane tape during the experiment. The measurements of reflectance spectra and OCT imaging of the acupoints and nonacupoints were measured before and at 2, 30, and 60 minutes after laser irradiated for duration of 15 minutes or 45 minutes, separately. Each measured point of a subject was irradiated only once a day, and other measured points of the subject were irradiated on the next day, and so on. The temperatures of acupoints and nonacupoints were measured in the corresponding time.

### 2.4. Statistical Analysis

Data from all samples were presented as means ± SD and analyzed using the SPSS 16.0 software paired-test. The *P* value < 0.05 indicated a significant difference.

## 3. Results

**Figure 3:**
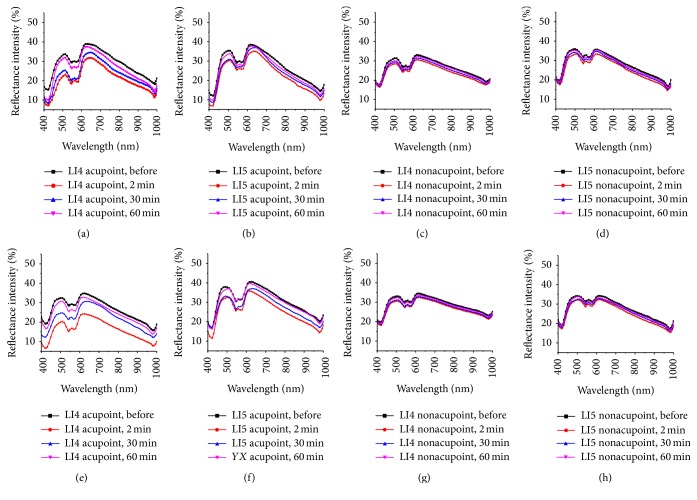
Reflectance spectra of the two acupoints and their nonacupoints before and 2, 30, and 60 minutes after the LI4 acupoint was irradiated by a 658 nm laser for 15 minutes and 45 minutes. The top row shows that LI4 acupoint was irradiated for 15 minutes; the bottom row shows that LI4 acupoint was irradiated for 45 minutes: (a) and (e) LI4 acupoint; (b) and (f) LI5 acupoint; (c) and (g) LI4 nonacupoint; (d) and (h) LI5 nonacupoint.

Ten healthy subjects (5 males, 5 females) were tested in this study. All subjects were measured under the same experimental conditions, and measurement of each point (acupoint or nonacupoint) was repeated six times and averaged. In Figures 2 and 3, we can see that the shapes of the spectral curves for various measured points are similar, and the troughs of each curve at the wavelengths of 422, 544, 577, and 980 nm are not changed. All the curves descend to the lowest at 2 minutes after irradiation compared with the curve before irradiation. It means that the maximum reduction value of RIs existed at 2 minutes after the laser irradiation. Then the RIs of the measured spectra increased and almost rose to the same amplitudes at 60 min after the laser irradiation compared with the spectra curve before the irradiation.

Figure 3 shows the RIs among measured points, including the two acupoints on the same meridian and their corresponding reference nonacupoints, before and after the LI4 acupoint was irradiated by a 658 nm laser at 2, 30, and 60 min for durations of 15 and 45 minutes, respectively. To quantitatively analyze the RI characteristics of the two acupoints and their nonacupoints after the LI4 acupoint was irradiated for different durations (15 and 45 minutes), the maximum reduction values of their RIs at four troughs were evaluated and shown in Table 1. The maximum reduction values of RIs of the three acupoints after the LI4 acupoint with laser irradiation for duration of 45 minutes were larger than those after the LI4 acupoint was irradiated for 15 minutes, but the decrease of RIs of their nonacupoints was not remarkable.

**Figure 4 fig4:**
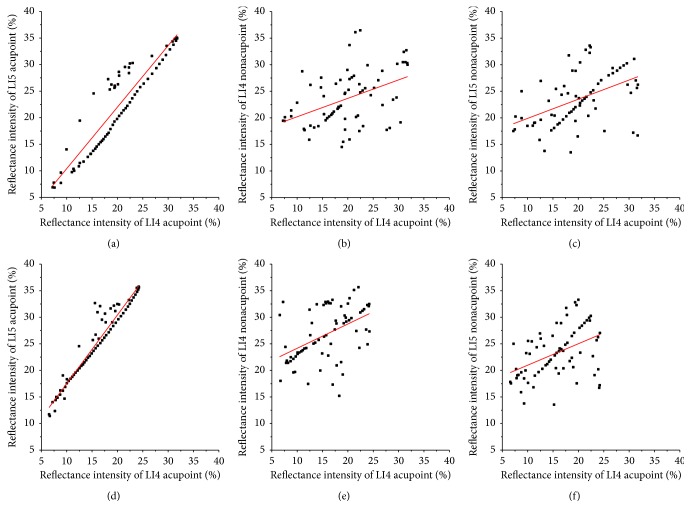
RIs of LI5 acupoint, LI4 nonacupoint, and LI5 nonacupoint versus RI of the LI4 acupoint after the LI4 acupoint was irradiated at 2 minutes, respectively. The top row shows that LI4 acupoint was irradiated for 15 minutes; the bottom row shows that LI4 acupoint was irradiated for 45 minutes: (a) *R* = 0.92, (b) *R* = 0.44, (c) *R* = 0.48, (d) *R* = 0.98, (e) *R* = 0.47, and (f) *R* = 0.41.

In the results in Figure 3, we calculated the correlation coefficient (*R*) between RI of the nonirradiated point and the irradiated acupoint after LI4 acupoint was irradiated for 15 minutes and 45 minutes at 2 minutes, as shown in Figure 4. After LI4 acupoint was irradiated for 15 or 45 minutes, *R* values between RI of the LI5 acupoint and RI of LI4 acupoint are 0.92 and 0.98, respectively. And the *P* values were <5%. While *R* values between RI of the LI4 nonacupoint and RI of LI4 acupoint are 0.44 and 0.47, *R* values between RI of the LI5 nonacupoint and RI of LI4 acupoint are 0.48 and 0.41, respectively. The *R* values indicate that there is a correlation between LI5 acupoint and LI4 acupoint, and the correlation between two nonacupoints and LI4 acupoint is very weak.

**Figure 5 fig5:**
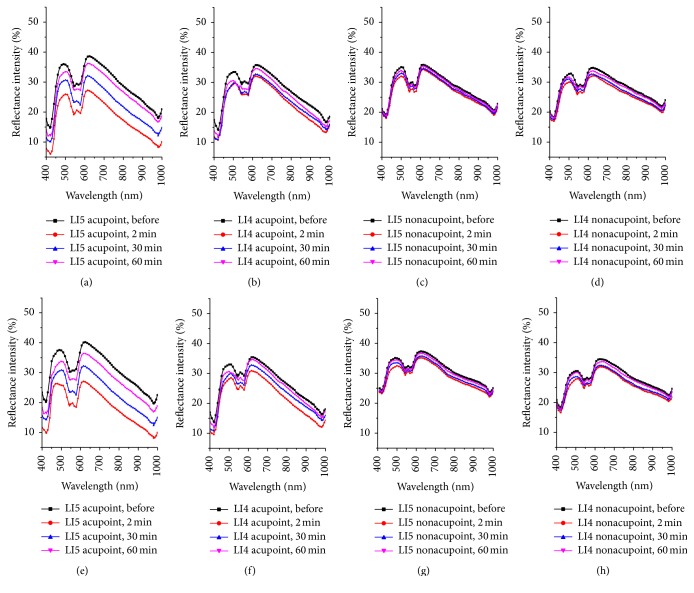
Reflectance spectra of the two acupoints and their nonacupoints before and 2, 30, and 60 minutes after the LI5 acupoint was irradiated by a 658 nm laser for 15 minutes and 45 minutes. The top row shows that LI5 acupoint was irradiated for 15 minutes; the bottom row shows that LI5 acupoint was irradiated for 45 minutes: (a) and (e) LI5 acupoint; (b) and (f) LI4 acupoint; (c) and (g) LI5 nonacupoint; (d) and (h) LI4 nonacupoint.

Figure 5 illustrates the dynamic changes of reflectance spectra of the LI4 and LI5 acupoints as a function of time elapsed before and after the LI5 acupoint was irradiated at 2, 30, and 60 minutes for 15 and 45 minutes, respectively. The RI change of the LI5 acupoint is more obvious than that of the LI4 acupoint after the LI5 acupoint was irradiated for 15 or 45 minutes at 2 minutes. The decreased values are shown in Table 2. Furthermore, the RI reduction values of the LI5 and LI4 acupoints after the LI5 acupoint was irradiated for 45 minutes were slightly larger than those after irradiation for 15 minutes.

**Figure 6 fig6:**
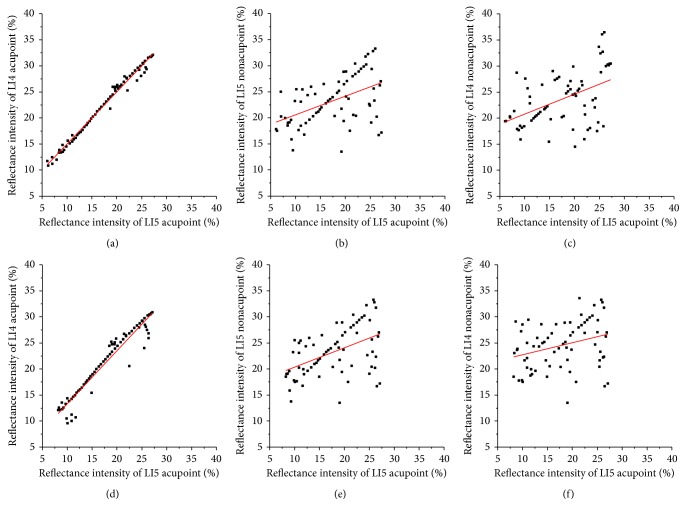
RIs of LI4 acupoint, LI5 nonacupoint, and LI4 nonacupoint versus RI of the LI5 acupoint after the LI5 acupoint was irradiated at 2 minutes, respectively. The top row shows that LI5 acupoint was irradiated for 15 minutes; the bottom row shows that LI5 acupoint was irradiated for 45 minutes: (a) *R* = 0.99, (b) *R* = 0.49, (c) *R* = 0.40, (d) *R* = 0.96, (e) *R* = 0.48, and (f) *R* = 0.31.

In the results in Figure 5, we calculated the correlation coefficient (*R*) between RI of the nonirradiated point and the irradiated acupoint after LI5 acupoint was irradiated for 15 minutes and 45 minutes at 2 minutes, as shown in Figure 6. After LI5 acupoint was irradiated of 15 or 45 minutes, *R* values between RI of the LI4 acupoint and RI of LI5 acupoint are 0.99 and 0.96, respectively. And *P* values were <5%. While *R* values between RI of the LI5 nonacupoint and RI of LI5 acupoint are 0.49 and 0.48, *R* values between RI of the LI4 nonacupoint and RI of LI5 acupoint are 0.40 and 0.31, respectively. Their *P* values were >5%. The results indicate that there is a correlation between LI4 acupoint and LI5 acupoint, and the strong correlation between two nonacupoints and LI5 acupoint is very weak.

**Figure 7 fig7:**
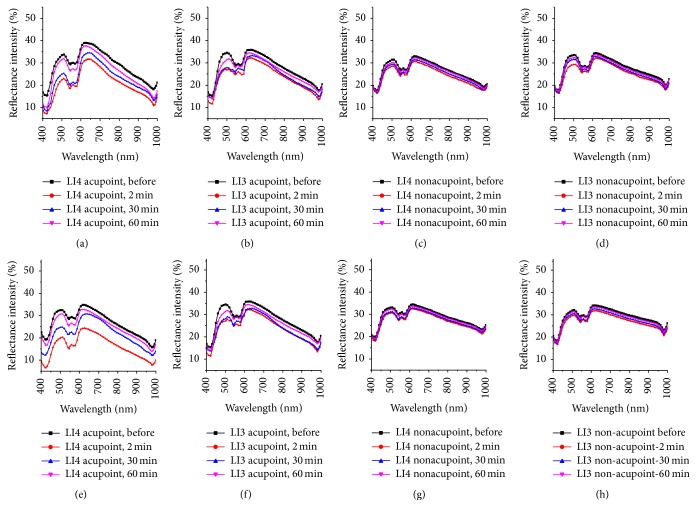
Reflectance spectra of the two acupoints and their nonacupoints before and 2, 30, and 60 minutes after the LI4 acupoint was irradiated by a 658 nm laser for 15 minutes and 45 minutes. The top row shows that LI4 acupoint was irradiated for 15 minutes; the bottom row shows that LI4 acupoint was irradiated for 45 minutes: (a) and (e) LI4 acupoint; (b) and (f) LI3 acupoint; (c) and (g) LI4 nonacupoint; (d) and (h) LI3 nonacupoint.

Figure 7 shows the RIs among measured points, including LI4 and LI3 acupoints on the same meridian and their corresponding reference nonacupoints, before and after the LI4 acupoint was irradiated by a 658 nm laser at 2, 30, and 60 min for durations of 15 and 45 minutes, respectively. To quantitatively analyze the RI characteristics of the two acupoints and their nonacupoints after the LI4 acupoint was irradiated for different durations (15 and 45 minutes), the maximum reduction values of their RIs at four troughs were evaluated and shown in Table 3. The maximum reduction values of RIs of the two acupoints after the LI4 acupoint with laser irradiation for duration of 45 minutes were larger than those after the LI4 acupoint was irradiated for 15 minutes, but the decrease of RIs of their nonacupoints was not remarkable.

**Figure 8 fig8:**
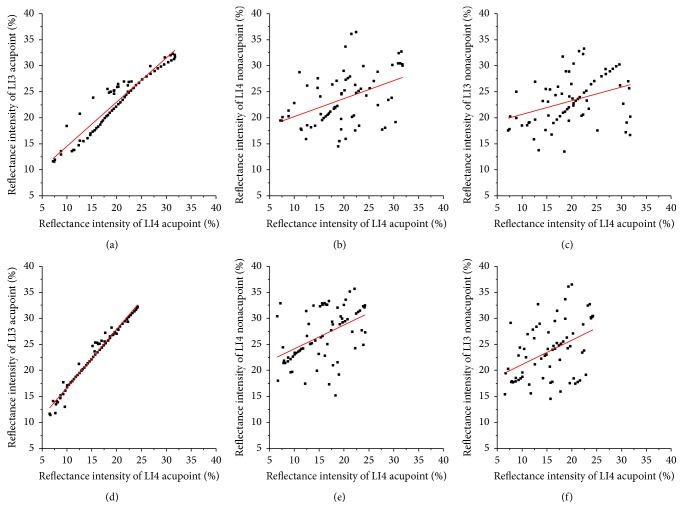
RIs of LI3 acupoint, LI4 nonacupoint, and LI3 nonacupoint versus RI of the LI4 acupoint after the LI4 acupoint was irradiated at 2 minutes, respectively. The top row shows that LI4 acupoint was irradiated for 15 minutes; the bottom row shows that LI4 acupoint was irradiated for 45 minutes: (a) *R* = 0.95, (b) *R* = 0.45, (c) *R* = 0.37, (d) *R* = 0.99, (e) *R* = 0.47, and (f) *R* = 0.42.

We calculated the correlation coefficient (*R*) between RI of the nonirradiated point and the irradiated acupoint after LI4 acupoint was irradiated for 15 minutes and 45 minutes at 2 minutes in accordance with Figure 7, as shown in Figure 8. After LI4 acupoint was irradiated of 15 or 45 minutes, *R* values between RI of the LI3 acupoint and RI of LI4 acupoint are 0.95 and 0.99, respectively. And *P* values were <5%. While *R* values between RI of the LI4 nonacupoint and RI of LI4 acupoint are 0.45 and 0.47, *R* values between RI of the LI3 nonacupoint and RI of LI4 acupoint are 0.37 and 0.42, respectively. Their *P* values were >5%. The results indicate that there is a correlation between LI3 acupoint and LI4 acupoint, and the strong correlation between two nonacupoints and LI4 acupoint is very weak.

**Figure 9 fig9:**
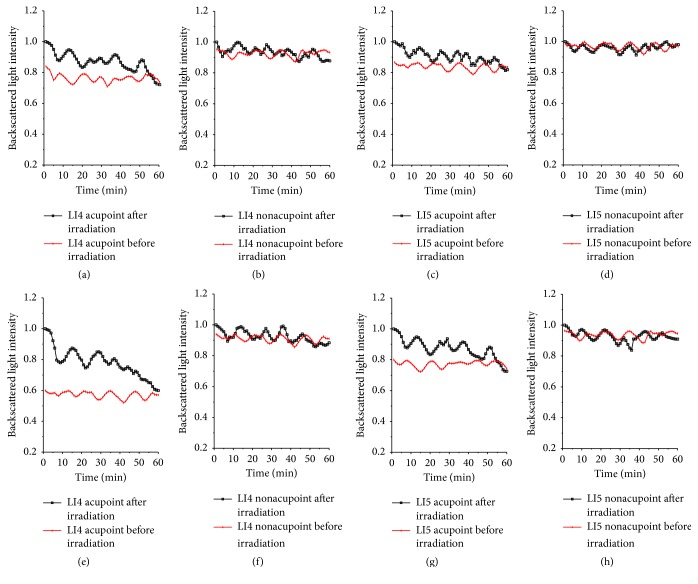
Backscattered light intensities of the LI4 and LI5 acupoints and their nonacupoints before and after the LI4 acupoint was irradiated by a 658 nm laser for 15 minutes and 45 minutes. The top row shows that LI4 acupoint was irradiated for 15 minutes; the bottom row shows that LI4 acupoint was irradiated for 45 minutes: (a) and (e) LI4 acupoint; (b) and (f) LI4 nonacupoint; (c) and (g) LI5 acupoint; (d) and (h) LI5 nonacupoint.

Figures 9 and 11 display the changes of normalized OCT backscattered light intensities of the LI4 and LI5 acupoints and their corresponding nonacupoints before and after the measured points were irradiated by the laser for duration of 15 minutes and 45 minutes at a depth of 0.3 mm under the surface of the skin, respectively. As we can see in Figure 4, all the OCT backscattered light intensities of the LI4 and LI5 acupoints and their nonacupoints before the LI4 acupoint was irradiated were kept stable, while after the LI4 acupoint was irradiated, the backscattered light intensities of the LI4 and LI5 acupoints increased prominently and the intensities of their nonacupoints were kept nearly stable. The backscattered light intensities of the LI4 and LI5 acupoints after the LI4 acupoint was irradiated for 15 minutes decreased from 1 to (0.73 ± 0.01) and (0.82 ± 0.01) (Figures 9(a) and 9(c)), respectively. The backscattered light intensities of the LI4 and LI5 acupoints separately decreased from 1 to (0.60 ± 0.01) and (0.72 ± 0.01) (Figures 9(e) and 9(g)) after LI4 acupoint was irradiated for 45 minutes. However, the values of the backscattered light intensities of the LI4 and LI5 nonacupoints almost remained unchanged during the experiments.

**Figure 10 fig10:**
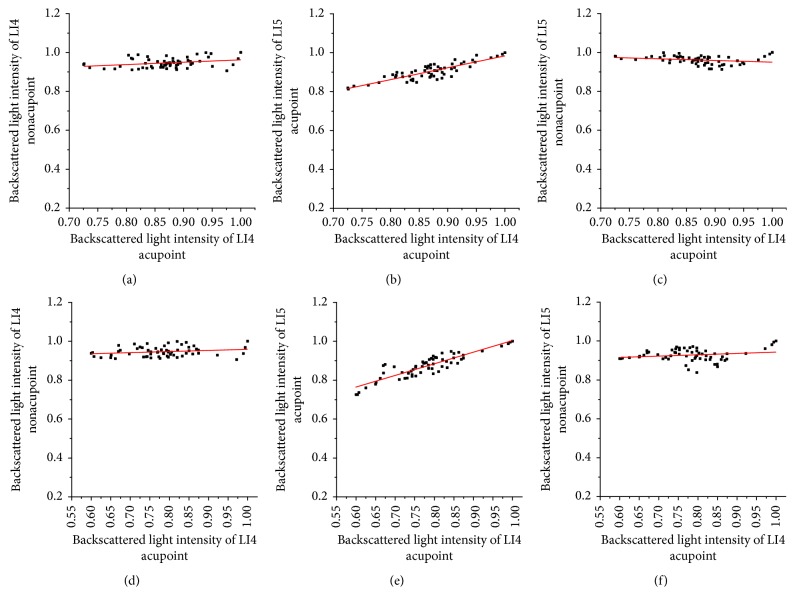
Backscattered light intensities of LI4 nonacupoint, LI5 acupoint, and LI5 nonacupoint versus backscattered light intensities of the LI4 acupoint after the LI4 acupoint was irradiated, respectively. The top row shows that LI4 acupoint was irradiated for 15 minutes; the bottom row shows that LI4 acupoint was irradiated for 45 minutes: (a) *R* = 0.29, (b) *R* = 0.86, (c) *R* = −0.25, (d) *R* = 0.21, (e) *R* = 0.89, and (f) *R* = 0.20.

We calculated the correlation coefficient (*R*) between backscattered light intensities of the nonirradiated point and the irradiated acupoint after LI4 acupoint was irradiated for 15 minutes and 45 minutes in accordance with Figure 9, as shown in Figure 10. After LI4 acupoint was irradiated for 15 or 45 minutes, *R* values between backscattered light intensities of the LI4 acupoint and backscattered light intensities of LI4 acupoint are 0.86 and 0.89, respectively. And *P* values were <5%. While *R* values between backscattered light intensities of the LI4 nonacupoint and RI of LI4 acupoint are 0.29 and 0.21, *R* values between backscattered light intensities of the LI5 nonacupoint and RI of LI4 acupoint are −0.25 and 0.20, respectively. Their *P* values were >5%. The results indicate that there is a correlation between LI5 acupoint and LI4 acupoint, and the strong correlation between two nonacupoints and LI4 acupoint is very weak.

**Figure 11 fig11:**
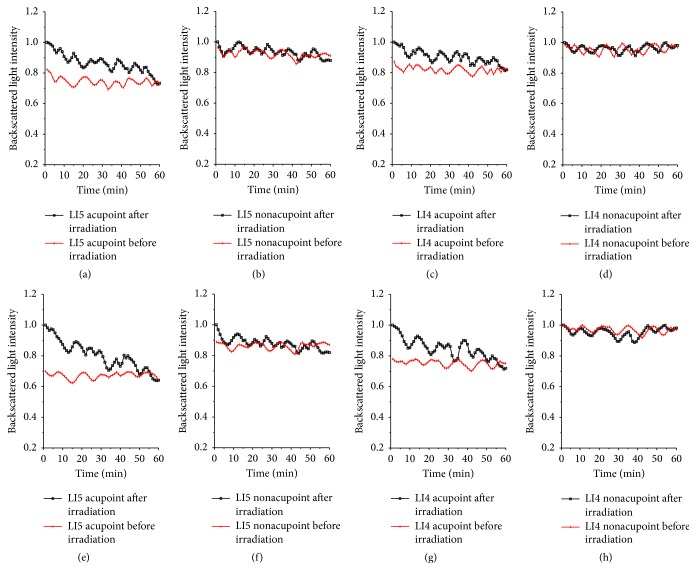
Backscattered light intensities of the LI5 and LI4 acupoints and their nonacupoints before and after the LI5 acupoint was irradiated by a 658 nm laser for 15 minutes and 45 minutes. The top row shows that LI5 acupoint was irradiated for 15 minutes; the bottom row shows that LI5 acupoint was irradiated for 45 minutes (a) and (e) LI5 acupoint; (b) and (f) LI5 nonacupoint; (c) and (g) LI4 acupoint; (d) and (h) LI4 nonacupoint.

As for the LI5 acupoint laser pretreatment, the values of backscattered light intensity of the LI5 and LI4 acupoints decreased from 1 to (0.73 ± 0.02) and (0.81 ± 0.02) after LI5 acupoint was irradiated for 15 minutes, separately, shown in Figures 6(a) and 6(c). The results of Figures 11(e) and 11(g) show that the values of the backscattered light intensities of the LI5 and LI4 acupoints changed from 1 to (0.64 ± 0.03) and (0.71 ± 0.03) for the LI5 acupoint with laser pretreatment for 45 minutes, respectively. But the values of the LI5 and LI4 nonacupoints almost remained unchanged, shown in Figure 11.

**Figure 12:**
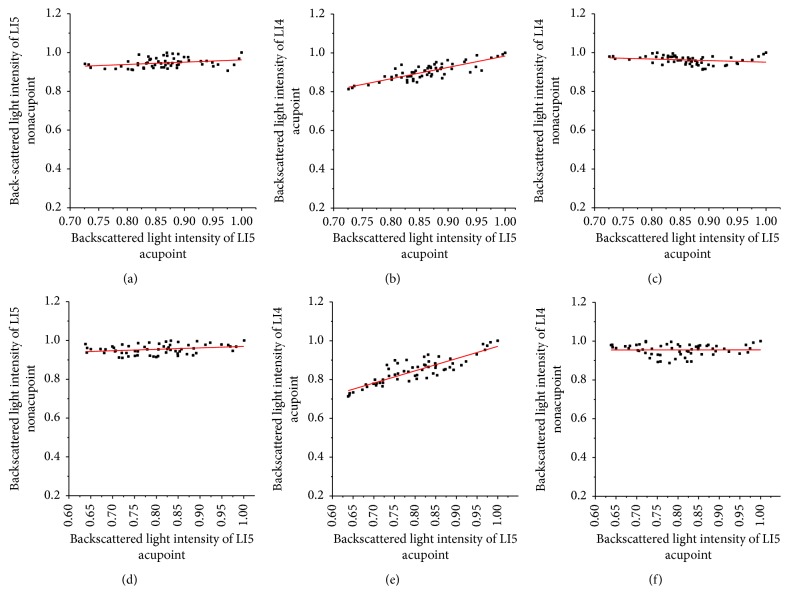
Backscattered light intensities of LI5 nonacupoint, LI4 acupoint, and LI4 nonacupoint versus backscattered light intensities of the LI5 acupoint after the LI4 acupoint was irradiated, respectively. The top row shows that LI5 acupoint was irradiated for 15 minutes; the bottom row shows that LI5 acupoint was irradiated for 45 minutes: (a) *R* = 0.27, (b) *R* = 0.83, (c) *R* = 0.23, (d) *R* = 0.28, (e) *R* = 0.87, and (f) *R* = 0.01.

We calculated the correlation coefficient (*R*) between backscattered light intensities of the nonirradiated point and the irradiated acupoint after LI5 acupoint was irradiated for 15 minutes and 45 minutes in accordance with Figure 11, as shown in Figure 12. After LI5 acupoint was irradiated of 15 or 45 minutes, *R* values between backscattered light intensities of the LI4 acupoint and backscattered light intensities of LI5 acupoint are 0.83 and 0.87, respectively. And *P* values were <5%. While *R* values between backscattered light intensities of the LI5 nonacupoint and RI of LI4 acupoint are 0.29 and 0.28, *R* values between backscattered light intensities of the LI4 nonacupoint and RI of LI5 acupoint are 0.23 and 0.01, respectively. Their *P* values were >5%. The results indicate that there is a correlation between LI4 acupoint and LI5 acupoint, and the strong correlation between two nonacupoints and LI5 acupoint is very weak.

## 4. Discussion

Searching for correlations between adjacent acupoints on the same meridian has an important significance. So, the changes of reflectance and backscattered light intensities of adjacent acupoints were monitored in this study. Since it is an optical method, it is noninvasive in contrast to other methods and hence is suitable for studying human acupoint.

In Figure 3, the reflectance spectra of the LI4, LI5 acupoints and their nonacupoints had significant changes after the LI4 acupoint was irradiated for some time, and the change of reflectance spectra of the LI4 acupoint was more obvious than the LI5 acupoint. Meanwhile, the reflectance spectra of the two nonacupoints almost remained unchanged during the experiments. The changes of reflectance spectra of the two acupoints and their nonacupoints after LI4 acupoint was irradiated for 45 minutes were consistent with those after the LI4 acupoint was irradiated for 15 minutes. However, the changes of reflectance spectra of two acupoints after LI4 was irradiated 45 minutes were more significant than those after LI4 acupoint was irradiated for 15 minutes. The reflectance spectra of the two acupoints also had prominent reductions, and the changes of reflectance spectra were significantly larger for the 45-minute exposure than for the 15-minute exposure, shown in Figure 5.

In Figure 7, the backscattered light intensity of the LI4 acupoint after the LI4 acupoint was irradiated had remarkably increased and then gradually came close to the initial value. The backscattered light intensity of the LI5 acupoint also had some changes, but not very distinctive. The changes of the backscattered light intensities of the LI4 and LI5 acupoints after LI4 acupoint was irradiated for 45 minutes were more obvious than for 15 minutes. However, the curves of the backscattered light intensities of the two nonacupoints were almost kept stable. Figure 9 was similar to Figure 7, indicating a similar situation which appeared after the LI5 acupoint was irradiated.

Comparing Figures 3 [Fig fig4] [Fig fig5] [Fig fig6] [Fig fig7] [Fig fig8] [Fig fig9] [Fig fig10] [Fig fig11]–12, the changes of reflectance spectra and backscattered light intensities were found to be related to the laser irradiation time to some extent, and all results of this study demonstrated that the LI4 acupoint is connected with the LI5 acupoint and LI3 acupoint. In other words, these results suggested that a correlation exists between adjacent acupoints on the same meridian to some extent.

## 5. Conclusions

In conclusion, this study presents a simple and noninvasive experimental method for studying the correlation in optical transport properties between the LI4, LI3, and LI5 acupoints on same-meridian* in vivo*. As to LI4 acupoint with laser irradiation for duration of 15 or 45 minutes, the OCT backscattered light intensities of LI4 and LI5 acupoints increased significantly, and the reflectance intensities (RIs) of the LI3, LI4, and LI5 acupoints decreased significantly. As to LI5 acupoint with laser irradiation for duration of 15 or 45 minutes, the changes of OCT backscattered light intensities of the corresponding irradiated acupoint and LI4 acupoint increased significantly, and the RIs decreased significantly. However, the OCT backscattered light intensities and RIs for their nonacupoints were almost not changed. Huang et al. [4] had studied the correlation between two adjacent acupuncture points during laser acupuncture by OCT. Our study supports their conclusion, and the conclusion is that a correlation exists between adjacent acupoints on the same meridian to some extent. In this study, we use diffuse reflectance spectra and OCT to monitor the changes of optical properties of acupoints and nonacupoints.

## Figures and Tables

**Table 1:** Decrease of RIs of the LI4, LI5 acupoints and their nonacupoints after the LI4 acupoint was irradiated for 15 and 45 minutes at 2 minutes, respectively.

	Irradiation time (15 minutes)				Irradiation time (45 minutes)			
	422.9 nm	544.0 nm	577.8 nm	980.0 nm	422.9 nm	544.0 nm	577.8 nm	980.0 nm
	Decrease of RI (%)				Decrease of RI (%)			
LI4	8.15	10.86	10.77	7.20	12.58	13.22	12.11	7.91
LI5	4.49	3.86	3.63	4.68	5.50	4.72	5.55	5.53
LI4 nonacupoint	1.37	2.50	2.53	1.31	1.71	2.49	2.59	1.57
LI5 nonacupoint	2.33	3.17	2.96	1.78	1.75	2.37	2.36	1.53

**Table 2:** Decrease of RIs of the LI5 and LI4 acupoints after the LI5 acupoint was irradiated for 15 and 45 minutes at 2 minutes, respectively.

	Irradiation time (15 minutes)				Irradiation time (45 minutes)			
	422.9 nm	544.0 nm	577.8 nm	980.0 nm	422.9 nm	544.0 nm	577.8 nm	980.0 nm
	Decrease of RI (%)				Decrease of RI (%)			
LI5	9.01	9.40	9.86	9.70	9.84	10.24	11.75	10.45
LI4	2.78	3.38	3.98	3.36	3.16	4.16	4.75	4.14
LI5 nonacupoint	1.28	2.27	2.06	2.07	0.94	1.95	1.89	1.26
LI4 nonacupoint	0.88	2.14	2.04	1.40	1.78	1.54	2.30	1.95

**Table 3:** Decrease of RIs of the LI4, LI3 acupoints and their nonacupoints after the LI4 acupoint was irradiated for 15 and 45 minutes at 2 minutes, respectively.

	Irradiation time (15 minutes)				Irradiation time (45 minutes)			
	422.9 nm	544.0 nm	577.8 nm	980.0 nm	422.9 nm	544.0 nm	577.8 nm	980.0 nm
	Decrease of RI (%)				Decrease of RI (%)			
LI4	8.15	10.86	10.77	7.20	12.58	13.22	12.11	7.91
LI3	3.75	3.94	4.79	3.94	4.65	4.05	5.21	4.53
LI4 nonacupoint	1.37	2.50	2.53	1.31	1.71	2.49	2.59	1.57
LI3 nonacupoint	1.48	2.27	2.43	1.80	1.84	2.40	2.18	1.85

## References

[1] Holden C. (1997). Thumbs up for acupuncture. *Science*.

[2] Gertsik G. Y., Zmievskoi G. N., Ivantsov V. I., Li S. M., Kim Y. B., Yun G. V. (2001). Optical methods for imaging acupuncture points and zones. *Biomedical Engineering*.

[3] Ueda Y., Hayashi K., Kuriowa K. (2005). The application of fMRI to basic experiments in acupuncture. The effects of stimulus points and content on cerebral activities and responses. *IEEE Engineering in Medicine and Biology Magazine*.

[4] Huang Y., Yang H., Wang Y., Zheng L., Xie S. Monitoring changes of optical attenuation coefficients of acupuncture points during laser acupuncture by optical coherence tomography.

[5] Yang G. J., Zhang E. L., Sheng Y. H. (2003). Comparison of therapeutic effects in treating 75 cases of neurocutaneous neuritis in thigh lateral with semiconductor laser irradiating acupoints and electro-acupuncture. *Laser Journal*.

[6] Hong W. X., Fan F. J., Song J. L. (2006). Comparison of therapeutic effects in treating neurodermatitis in laser-needleacupuncture and traditional acupuncture. *Laser Journal*.

[7] Hong W., Mo F. Z., Li J. Q. (2007). Observation on the comparison in therapeutic effects between the 650 nm laser acupoint irradiation and acupuncture on the treatment of benign prostatic hyperplasia. *Acta Laser Biology Sinica*.

[8] Streng A. (2007). Summary of the randomized controlled trials from the German model projects on acupuncture for chronic pain. *Journal of Chinese Medicine*.

[9] Zhang Y. (1993). *Clinical Application of Laser Acupuncture Therapy*.

[10] Ming L., Xi Z. Z. (2006). Laser acupuncture theory and light dosage selection. *Acta Laser Biology Sinica*.

[11] Whittaker P. (2004). Laser acupuncture: past, present, and future. *Lasers in Medical Science*.

[12] Zalewska-Kaszubska J., Obzejta D. (2004). Use of low-energy laser as adjunct treatment of alcohol addiction. *Lasers in Medical Science*.

[13] Litscher G., Rachbauer D., Ropele S. (2004). Acupuncture using laser needles modulates brain function: First evidence from functional transcranial Doppler sonography and functional magnetic resonance imaging. *Lasers in Medical Science*.

[14] Wong W., Xiao S. J., Ip W. Y., Guo X. Effects of a laser acupuncture therapy on treating pain.

[15] Jiao J. L., Liu T. C. Y., Li C. Z. (2003). Information biology of laser acupuncture. *Photonics and Imaging in Biology and Medicine*.

[16] Tsuei J. J. (1996). The science of acupuncture—theory and practice. *IEEE Engineering in Medicine and Biology Magazine*.

[17] Prokhorov E. F., González-Hernández J., Vorobiev Y. V., Morales-Sánchez E., Prokhorova T. E., Lelo de Larrea G. Z. (2000). In vivo electrical characteristics of human skin, including at biological active points. *Medical and Biological Engineering and Computing*.

[18] Ding G. H., Yao W., Chu J. H. (2001). Spectral characteristic of infrared radiations of some acupoint and non-acupoint areas in human arm surface. *Chinese Science Bulletin*.

[19] Lo S.-Y. (2002). Meridians in acupuncture and infrared imaging. *Medical Hypotheses*.

[20] Yang H.-Q., Xie S.-S., Liu S.-H., Li H., Guo Z.-Y. (2007). Differences in optical transport properties between human meridian and non-meridian. *The American Journal of Chinese Medicine*.

[21] Chen G.-Z., Xu Y.-X., Wang Y.-H. (2011). Optical transport properties along the pericardium meridian under different pressure. *Journal of Lasers in Medical Sciences*.

[22] Jiang X.-H., Liu H.-P., Guo Z.-Y., Meng Y.-Y., Zeng C.-C., Liu S.-H. (2010). Comparative study of reflectance spectroscopy of women's acupoints around menstruation. *Spectroscopy and Spectral Analysis*.

[23] Zhong H. Q., Zhang Z. D., Guo Z. Y. (2010). Using OCT image to distinguish human acupoint from non-acupoint tissues after irradiation with laser in vivo:a pilot study. *Chinese Optics Letters*.

[24] Shen Y. F., Younger J., Goddard G., Mackey S. (2009). Randomized clinical trial of acupuncture for myofascial pain of the jaw muscles. *Journal of Orofacial Pain*.

[25] Schiapparelli P., Allais G., Rolando S. (2011). Acupuncture in primary headache treatment. *Neurological Sciences*.

[26] Zhou L. P., Wu G. Y., Wei H. J. (2015). Effects of titanium dioxide nanoparticles coupled with diode laser on optical properties of in vitro normal and cancerous human lung tissues studied with optical coherence tomography and diffuse reflectance spectra. *Journal of Biomedical Optics*.

[27] Asher G. N., Motsinger-Reif A. A., Jonas D. E., Viera A. J. (2011). Quality of reporting on randomised controlled trials of auriculotherapy for pain. *Acupuncture in Medicine*.

